# Hazard and cumulative incidence of umbilical cord metabolic acidemia at birth in fetuses experiencing the second stage of labor and pathologic intrapartum fetal heart rate requiring expedited delivery

**DOI:** 10.1007/s00404-022-06594-1

**Published:** 2022-05-21

**Authors:** Paolo Ivo Cavoretto, Anna Seidenari, Antonio Farina

**Affiliations:** 1 Gynecology and Obstetrics Department, IRCCS San Raffaele Hospital, University Vita-Salute, Milan, Italy; 2 Division of Obstetrics and Prenatal Medicine, Department of Medicine and Surgery (DIMEC), IRCCS Azienda Ospedaliero-Universitaria di Bologna, Italy, University of Bologna, Via Massarenti 13, 40138 Bologna, Italy

**Keywords:** Childbirth, Fetal surveillance, Umbilical cord pH, Metabolic acidemia, International Federation of Gynecology and Obstetrics 2015 cardiotocography classification, Intrapartum fetal monitoring, Survival model, Risk estimation, Cardiotocography

## Abstract

**Purpose:**

The aim of the study was to determine the cause-specific hazard (CSH) and the cumulative incidence function (CIF) for umbilical cord metabolic acidemia at birth (MA; pH < 7.0 and/or BE $\le$ − 12 mmol/L) at delivery in patients experiencing the 2nd stage of labor (2STG), stratified for both FIGO-2015 pathologic intrapartum cardiotocography requiring expedited delivery (CTG_RED) and duration of 2nd stage of labor.

**Methods:**

3459 pregnancies experiencing the 2nd stage of labor and delivering at the Division of Obstetrics and Prenatal Medicine, IRCCS Sant’Orsola-Malpighi Hospital, Bologna (Italy), were identified between 2018 and 2019. Survival analysis was used to assess CSH and CIF for MA, stratified for FIGO-2015 pathologic CTG and relevant covariates.

**Results:**

FIGO-2015 pathological CTG with expedited operative delivery or urgent cesarean section within 10 or 20 min from diagnosis, respectively occurred in 282/3459 (8.20%). The rate of MA at delivery was 3.32% (115/3459). The spline of CSH for MA showed a direct correlation with the duration of 2STG always presenting higher values and greater slope in the presence of pathologic CTG, with plateau between 60 and 120 min and rapid increase after 120 min. The CIF at 180 min in the 2STG was 2.67% for nonpathological and 10.63% for pathological CTG_RED. Nulliparity, pathological CTG, and meconium-stained amniotic fluid resulted significant predictors of MA in our multivariable model.

**Conclusion:**

The risk for MA increases moderately across the 2STG with nonpathological CTG and quadruples with pathological CTG_RED. Adjustment for other predictors of MA including meconium-stained amniotic fluid and nulliparity reveals a significant hazard increase for MA associated with pathologic CTG_RED.

**Supplementary Information:**

The online version contains supplementary material available at 10.1007/s00404-022-06594-1.

## Background

Intrapartum fetal hypoxia determines lactic acid accumulation, potentially leading to umbilical cord metabolic acidemia at birth (MA) and impairment of the fetal heart rate (FHR) vagal regulation [1, 2]. It was widely demonstrated that the 2nd stage of labor (2STG) presents the highest risk of MA since, with advancing labor, the fetus employs part of its bases reserves, achieving progressively a lower tolerance to hypoxic insults [3–5]. In fact, fetal scalp pH during normal labor decreases 0.016 pH unit per hour in the 1st stage and 0.11–0.12 pH unit per hour in the 2STG, showing a clear trend towards higher bases depletion from the 1st to the 2STG [6]. Consequences of MA result in major medical, psychological, social, and medicolegal implications with profound involvement of individual families and entire society. [7]

Different scientific Societies proposed intrapartum CTG classifications to detect and to prevent MA [8–10]. However, duration of labor was not considered with the exception of some previous paper coming from our group in Bologna [11], and the associated risk of MA remains empirically estimated.

## Objective

The aim of this study was to determine the cause-specific hazard (CSH) and the cumulative incidence function (CIF) for MA (pH < 7.0 and/or BE $\le$ − 12 mmol/L) in the 2STG stratified for FIGO-2015 pathologic intrapartum CTG requiring expedited delivery (CTG_RED) and adjusted for several clinically relevant covariates, within the multivariable model.

## Methods

### Study design and setting

This was a retrospective study collecting deliveries in the years 2018–2019 at the Division of Obstetrics and Prenatal Medicine Department of Medicine and Surgery (DIMEC), IRCCS Sant’Orsola-Malpighi Hospital, University of Bologna, Italy. The study was presented according to the Strengthening the Reporting of Observational Studies in Epidemiology (STROBE) statement guidelines for reporting observational studies. [12].

### Participants

Singleton pregnancies in labor at 38^+0^ to 41^+3^ gestational weeks, monitored with continuous CTG tracings, recording fetal heart rate (FHR) and simultaneous maternal heart frequency, were identified. Only those patients experiencing the 2STG were included. Exclusion criteria were: cesarean section performed at the 1st stage of labor, nonvertex presentations, intrapartum fetal deaths, twin or multifetal pregnancies, pregnancies in which the fetus was affected by chromosomal defects, genetic syndromes, cardiac arrhythmias or a major structural malformations, absence of funicular pH measurement within 5 min from birth. Maternal health conditions were not considered as exclusion criteria. The electronic medical charts of patients were retrospectively reviewed. Supplemental Fig. 1 shows the STROBE flowchart of patients’ selection.

### Variables

All included patients underwent continuous CTG monitoring in the 1st and 2STG and rapid funicular hemogasanalysis with umbilical artery pH measurement at birth. Onset of 2STG was outlined at 10 cm of cervical dilatation or in presence of desire to bear down [13]. Umbilical cord metabolic acidemia at birth (MA) was defined with umbilical artery pH < 7.0 and/or BE ≤ − 12 mmol/L.[14].

The CTG traces were stored electronically for offline analysis at 1 cm/min paper speed. Other clinically relevant covariates including maternal age, parity, BMI, meconium-stained amniotic fluid, induction of labor with prostaglandins and neonatal weight, neuraxial analgesia, gestational age at delivery and mode of delivery, were added to the multivariable model. Meconium-stained amniotic fluid was classified as following: Grade I—meconium-stained amniotic fluid is translucent, light yellow-green in color. Grade II—meconium-stained amniotic fluid is opalescent with deep green and light yellow in color. Grade III—meconium-stained amniotic fluid is opaque and deep green in color. The presence (grade II or III) or absence (grade 0 or I) of meconium-stained amniotic fluid was evaluated with dichotomic categorization at the time of delivery by visual inspection of attending physician, consistently with standard clinical practice. Birthweight (BW) was transformed into corresponding centile according to Intergrowth-21st internationally recognized reference standards [15], and neonates were categorized in small for gestational age (SGA, BW < 10th centile); fetal growth restriction (FGR, BW < 3rd centile), large for gestational age (LGA, BW > 90th centile) or macrosomia (BW > 97th centile).

### Data sources and CTG assessment

Umbilical cord blood was analyzed within 5 min from delivery using automated analyzers placed in the labor ward (Bayer RAPIDLAB 865; Diamond Diagnostics, Holliston, MA, USA). In all cases, particularly with suspected metabolic acidemia, a quick cord milking is generally carried out at our center and the cord is clamped immediately. An anonymized database was produced including qualitative and quantitative descriptions of variables related to the FHR abnormalities object of this study, according to FIGO-2015 (type of CTG abnormality, duration, mean FHR, lowest FHR). Extent of the 2STG was recorded. CTG was carried out with available monitors (Avalon FM30 or FM50 Philips Healthcare, Netherlands; Sonicaid FM800 or TEAM IP, Huntleigh Arjo Inc., Addison IL, USA) equipped with ultrasound transducers applied on maternal abdomen or on the fetal skull, as appropriate. Both fetal and maternal heart rates tracings were recorded in all cases in order to exclude wrong signal sampling.

The CTG traces and relative FHR patterns were assessed and classified by the obstetrician on call. All diagnoses of both pathological CTG and pathological CTG_RED were double-checked and confirmed by one of the coauthors, with discussion in case of uncertain diagnosis. The CTG tracings were categorized according to the FIGO-2015 criteria. Decelerations > 5 min or bradycardia > 10 min were diagnosed when FHR was persistently below 80 beats per min (bpm) with reduced variability within the deceleration. Normal variability was defined as bandwidth amplitude of 5 − 25 bpm (reduced variability when below 5 bpm) [8]. Additional description on CTG methodology can be obtained from previous studies from our group [11]. Pathological CTG was defined according to FIGO-2015 criteria (baseline < 100 bpm; abnormal variability or sinusoidal pattern; repetitive late or prolonged decelerations during > 30 min or 20 min if reduced variability, or one prolonged deceleration with > 5 min). After diagnosis of pathological CTG immediate action to correct reversible causes was carried out. In case of tachysystole or excessive contractions the action was reducing or stopping oxytocin infusion, removing administered prostaglandins if possible, and/or starting acute tocolysis with beta-adrenergic agonists or atosiban; in case of suspected cord compression the mother was asked to stop pushing, she was turned on her side or placed in another position; in case of acute maternal hypotension due to neuraxial analgesia rapid fluid administration and/or an intravenous ephedrine bolus was undertaken. If these actions were not possible or successful within a limited period or if acute conditions were diagnosed (cord prolapse, uterine rupture, or placental abruption) requirement for immediate delivery (RED) was established and immediate expedited delivery was achieved. Time between diagnosis of CTG_RED and delivery was kept below 20 min in all cases in this study.

## Statistical analysis

Demographic data were analyzed by routine test including Student’s *t* test, Mann–Whitney *U* test and *χ*^2^ test. The evaluation of the risk of fetal–neonatal MA was based the survival model for the time of delivery with MA stratified according with appearance of FIGO-2015 pathologic CTG_RED. The analysis focuses on observable quantities such as the cause-specific hazard (CSH) and the cumulative incidence (CIF). The CIF calculated by the Aalen–Johansen estimator describes the incidence of the occurrence of the event of interest throughout the 2STG.

Along with the CIF for MA, the CSH was calculated. The CSH can be defined as the instantaneous rate of occurrence of a given event among the patients still event-free during the 2STG. The CSH provides additional useful insight over and above what can be provided from the CIF. It can be used to obtain more detailed information about the instantaneous event rate for the overall group of patients or when comparing groups. The instantaneous nature of CSH suggests that it responds more quickly to changes in risk associated with the event under investigation. CSH and CIF were both stratified according with the major variable of interest (i.e. the presence or absence of a pathologic CTG_RED). Multivariable analysis was conducted according with the Cox regression providing the hazard ratio (HR), which measures the relationship between the candidate predictive factors and the outcome. Proportional hazard assumption of the significant predictors was also tested. Statistical significance was considered when *p* value < 0.05 with a two-tailed testing. Stata vers. 17 was used for all statistical analyses.

## Results

**Fig. 1:**
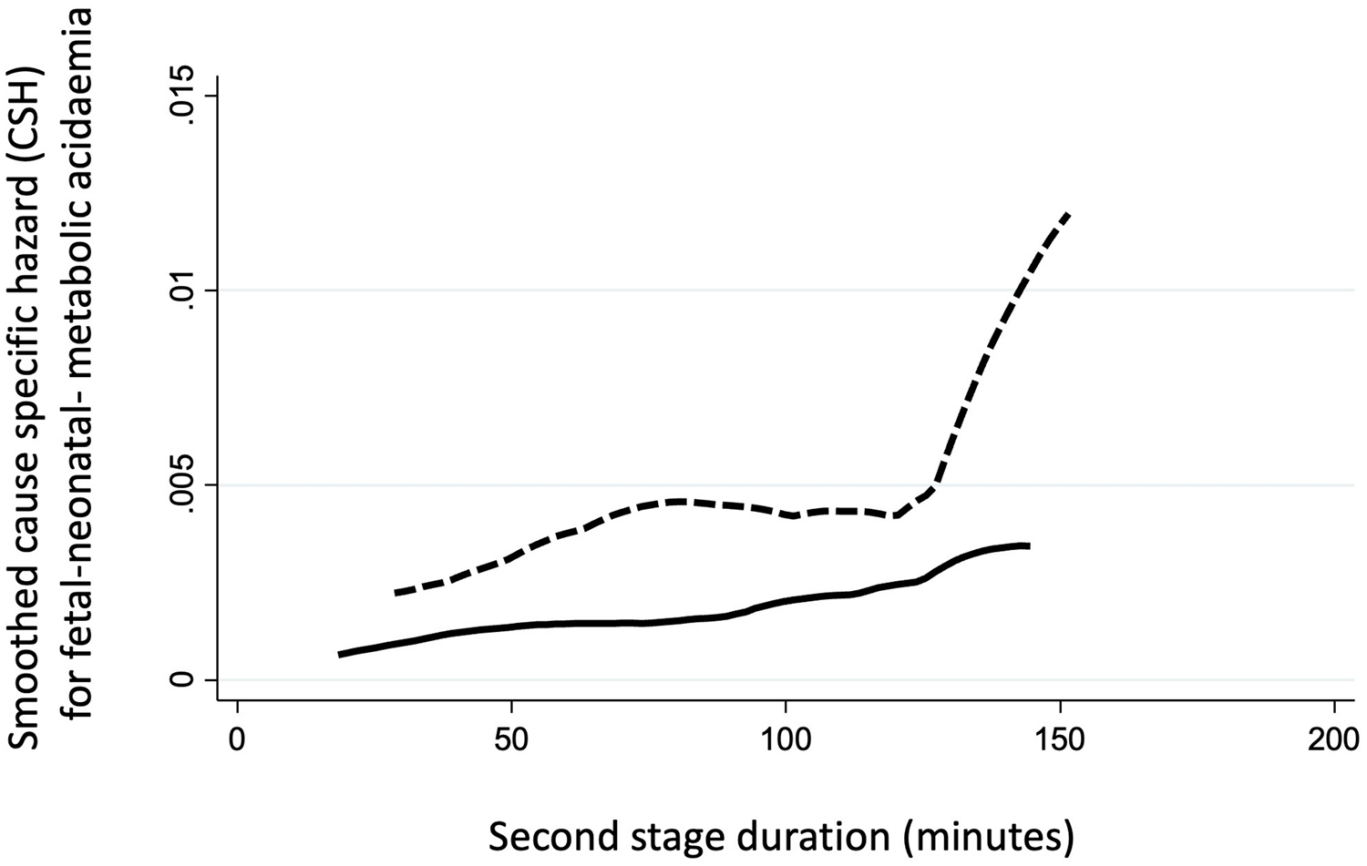
Kernel-based estimates of the cause-specific hazard (CSH) functions for umbilical cord metabolic acidemia at birth according to CTG. As shown, FIGO-2015 pathologic CTG requiring expedited delivery (dotted line) is associated with higher CSH and greater increase per unit of time when compared with nonpathologic CTG (solid line)

Pregnancies retrospectively identified were 3459: all experienced the 2STG and delivered at Sant’Orsola Malpighi Hospital (Bologna, Italy). There were 282 (8.2%) pathologic CTG_RED and the incidence of MA was 3.32%. The available demographic data are reported in Table 1. As shown, higher rate of nulliparity, meconium-stained amniotic fluid was found for the group with pH < 7.0 and/or BE ≤ − 12 mmol/L. Duration of labor (both total and 2nd stage only) was also higher in acidemic neonates versus nonacidemic. A higher proportion of vacuum deliveries and a lower proportion of spontaneous deliveries occurred in acidemic versus nonacidemic neonates. APGAR 1′ and 5′ were also lower in acidemic neonates. No significant difference was found for all other variables analyzed. Sixty-six percent (186/282) of CTG_RED cases presented initially with a suspect CTG. In MA cases, bradycardia ≥ 10 min was present in 26.7% of the cases (8/30); in neonates with normal pH the bradycardia ≥ 10 min was instead present only in 3.2% (8/252) of the cases (*p* value < 0.001). Late recurrent or prolonged decelerations for > 30 min (or > 20 min if reduced variability) were present in 73.3% of the MA cases (22/30) and in 88.1% (222/252) of the neonates with normal pH (*p* value < 0.001). Figure 1 reports the kernel-based estimates of CSH of MA stratified according with CTG category (pathologic CTG_RED or nonpathologic CTG). In the presence of FIGO-2015 pathologic CTG_RED, for a fixed duration of the second stage, CSH is significantly higher, with greater slope and intercept. Interestingly, the CSH of the group including nonpathological CTG_RED shows a nearly linear direct relationship between smoothed hazard estimates of MA and labor duration (Fig. 1, solid line), whereas that of pathological CTG_REDs show a faster risk acceleration above 120 min (Fig. 1, dotted line) after displaying a plateau between 60 and 120 min.

**Table 1:** Demographic variables, characteristics of the patients, pregnancies, and deliveries included in the study; the data are expressed as mean ± SD or percentage or median (interquartile range)

	pH < 7.00 and/or BE excess ≤ − 12 mmol/L (*n* = 115)	pH ≥ 7.00 and/or BE excess > − 12 mmol/L (*n* = 3344)	*p* value
Mother			
Maternal age (years)	33.1 ± 5.28	33.2 ± 5.44	0.865
BMI	22.75 ± 3.77	22.81 ± 4.19	0.883
Nulliparity (%)	87.0	55.1	< 0.001
Miscarriages (> 2)	0	1.4	0.200
Time from the last delivery (years)	2.16	2.35	0.261
Fetus			
Neonatal weight	3297 ± 467	3294 ± 467	0.069
SGA < 10 centile	5.2	6.9	0.477
FGR < 3 centile	0	1.4	0.200
LGA > 90 centile	11.3	10.5	0.787
Macrosomia > 97 centile	5.2	4.0	0.522
BE excess mmol/L	− 14 ± 1.87	− 5 ± 3.59	–
APGAR 1	7 (1–9)	9 (6–10)	< 0.001
APGAR 5	8 (6–10)	10 (8–10)	< 0.001
Delivery and procedures			
Gestational age at delivery (weeks)	39 ± 1.78	39 ± 2.00	0.363
Induction of labor total (%)	50.4	42.9	0.109
Neuraxial analgesia (%)	41.7	35.9	0.204
Meconium-stained amniotic fluid (%)	29.6	11.1	< 0.001
Duration of labor (min)	302 (111–391)	161 (65–280)	< 0.001
Duration of the 2nd stage labor (min)	41 (28–62.5)	23 (12–41)	< 0.001
CTG_RED (%)	26.1	7.5	< 0.001
Mode of delivery			
Spontaneous (%)	11.4	89.7	< 0.001
Vacuum (%)	71.3	6.3	< 0.001
Cesarean delivery (%)	17.3	4.0	< 0.001

*BMI* body mass index, *SGA* neonate small for gestational age (BW < 10 centile), *FGR* neonate with fetal growth restriction (BW < 3 centile), *LGA* neonate large for gestational age (BW > 90 centile) or neonatal macrosomia (BW > 97 centile), *CTG_RED* intrapartum CTG requiring expedited delivery

**Fig. 2:**
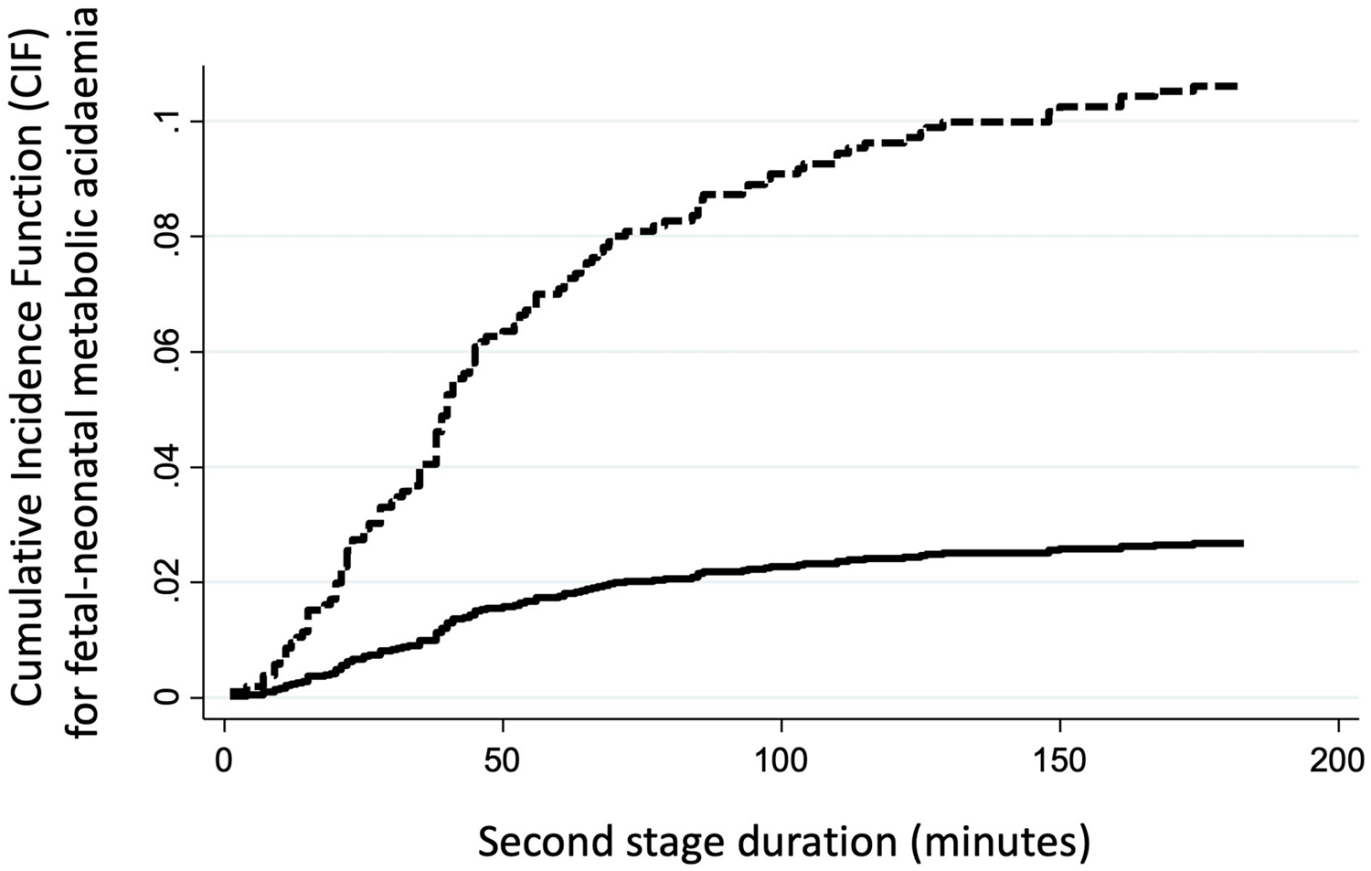
Cumulative incidence functions (CIF) for umbilical cord metabolic acidemia at birth according to presence (dotted line) or absence (solid line) of pathological CTG requiring expedited delivery

Figure 2 shows the CIFs for MA, with FIGO-2015 nonpathological or pathological CTG_RED in relation to 2STG duration. As shown, in the presence of a pathological CTG_RED the risk of MA increases about fourfold. In fact, at 180 min, the CIF for MA is 10.63% vs 2.67% in the presence of pathologic CTG_RED or nonpathologic CTG, respectively (*p* value < 0.001).

In the multivariable analysis the assumption of proportional hazard was not violated (a borderline p value showing a time-dependent effect of CTG_RED after 120 min was however detected). The statistically significant independent risk factors for prediction of MA were: nulliparity, meconium-stained amniotic fluid and FIGO-2015 pathological CTG_RED, the latter being the most important predictor. Assuming a proportional hazard model the quoted HR value for pathologic CTG_RED was 2.31 (Table 2). The effect of all other relevant covariates of interest tested was not statistically significant within the model at the predefined level of significance.

**Table 2:** Output of the Cox regression model obtained for 3459 observations with dependent variable umbilical cord metabolic acidemia at birth (pH < 7.00 and/or base excess ≤ − 12 mmol/L

	HR	Standard Error	*p* value	95% confidence intervals	
				Lower bound	Upper bound
Nulliparity	1.517	0.190	0.028	1.045	2.204
Meconium-stained amniotic fluid	2.178	0.210	< 0.001	1.443	3.286
CTG_RED	2.307	0.218	< 0.001	1.506	3.534

*HR* hazard ratio, *CTG_RED* pathological intrapartum CTG requiring expedited delivery

## Discussion

### Main findings

This was the first study undertaking a multivariable survival model of MA occurring at the 2STG. The research showed that, firstly, instantaneous CSH of MA increases overtime in the 2STG irrespectively of the presence of pathologic CTG_RED. Secondly, instantaneous CSH is substantially greater in cases with FIGO-2015 pathologic CTG_RED as compared to cases with nonpathological CTG. Thirdly, in cases with FIGO-2015 pathological CTG_RED the CSH showed a nonlinear pattern, having a plateau between 60 to 120 min and a rapid increase after 120 min in the 2STG. While in the absence of pathologic CTG, a linear trend was observed, the group with FIGO-2015 pathological CTG_REDs seems to have a bimodal trend. In fact, at the beginning of the 2STG there is a first acceleration reaching a plateau at 60 min and then return with greater evidence after 120 min. The acceleration of CSH observed in the pathological CTG_RED group after 2 h from the beginning of the 2STG reflects the phase of fetal maladaptation, becoming evident after that of fetal compensation occurring earlier in the 2STG.

Finally, the CIF for MA reaches 2.7% and 10.6% at 180 min in the 2STG for low-risk (nonpathological CTG) or high-risk (pathological CTG_RED) cases, respectively. Therefore, there was a fourfold risk increase due to CTG categorization and after adjustment for covariates the hazard further increases more than twofold (HR = 2.31).

### Strengths and limitations

This is the first paper that used a survival-based method to quantify the risk of MA. Clinicians managing the delivery room may use this statistical analysis in a prospective fashion for their data examination, in order to have an instantaneous estimate of risk for each individual fetus.

A limitation of this study is that CTG classification remains a hard challenge since there is a great interobserver variability with interpretation of the traces [16] and evidence for the impact of CTG training on neonatal and maternal outcomes are somehow limited [17]. The previous research demonstrated a more severe retrospective classification of decelerations and variability of intrapartum CTG with knowledge of adverse neonatal outcome [18, 19]. More so, in this retrospective study some data was missing (such as estimated fetal weight) and the decision to proceed with an expedited delivery is a function of the experience of the operator and facilities available at each local setting. Finally, only CTG traces defined as CTG_RED by attending obstetricians were reviewed; this may introduce bias of unknown degree.

### Interpretation

The finding of an acceleration of CSH according with greater duration of the 2STG and presence of FIGO-2015 pathological CTGs is in line with previous observations, where advanced phases of the 2STG, bear a greater risk of MA and hypoxia (pH ≤ 7.10), as compared to early stages [11]. Our current findings are also in agreement with the previous observation that majority of fetuses are not acidemic, even when the CTG trace is pathological (252/282 or 89.4% in our series) [20]. Clinicians should take into account the described impact of pathological CTG time dependency for interpretation and risk estimation of MA. Clinical management including either conservative approaches, or obstetric interventions accelerating delivery, should be tailored based upon multivariable individual patients risks rather than on a mere interpretation of the CTG tracings.

Deceleration area was shown as the most predictive electronic fetal monitoring pattern for MA [21], however this was not deemed to be the resolution of the prediction problem, given the diverse etiology of decelerations [22]. FIGO-2015 criteria take instead into account such etiologic differentiation [8]. In this paper, the etiology of each FIGO-2015 specific feature was not considered, instead the main outcome was the presence of a pathologic CTG requiring expedited delivery. A novel survival-based approach was employed, to calculate the CSH and the CIF of MA. This approach gives a better estimation of the episode of interest. Again, the CSH stratified according with labor duration occurrence of FIGO-2015 pathologic CTG_RED and meconium-stained amniotic fluid was never reported in the literature.

This study confirmed an expected finding given by the association of FIGO-2015 pathologic CTG_RED with a higher risk of MA and presented the novel finding of instantaneous risk as a function of both CTG categorization and labor duration. Surprisingly, little is reported in the literature about risk quantification of abnormal CTG alone or combined with other factors, such as extent of 2STG. Our group recently presented a logistic regression-based method to calculate the risk for MA (pH ≤ 7.10) using the baseline risk of the general population combined with that associated to major features of FIGO-2015 pathologic CTG. The CTG features included were bradycardia > 10 min, decelerations > 5 min and repetitive (associated with > 50% contractions), prolonged or late decelerations > 30 min [11]. As expected, the combination of more CTG anomalies was associated with the highest risk of MA [23, 24], however the risk was higher for increasing durations of the second stage of labor.

## Conclusions

Despite the worldwide diffusion of intrapartum CTG, risk quantification of a major event of interest such as MA is poorly described in the literature. This study is a further step forward towards individualization of intrapartum care. In fact, risks of MA promoting obstetrics interventions to expedited delivery should be assessed within more complex algorithms, including at least duration of the 2STG and possibly other covariates, rather than on the mere observation of CTG tracings. We have also shown that nonpathological CTG_RED presents increasing risk of MA, with progression of the 2STG. This is in line with the observation that fetal scalp pH declines with advancing labor due to bases depletion occurring in subacute hypoxic conditions [6]. It is not surprising that meconium-stained amniotic fluid represents a major risk factors in view of its role in intra-amniotic and fetal inflammation as well as a risk factor neonatal morbidity [13, 25].

Given the nature of this research area, it is unlikely that high-level evidence would be produced with randomized trials. Moreover, the knowledge produced in this study concerning the additive effect of CTG category and second stage duration was shown before with different methodology on two different dataset [11].

Several research questions remain unanswered, including the effect on the statistical model of other clinically relevant covariates both on maternal and fetal side. In particular, for the fetal component would be interesting to assess more the risk of MA in relation to intrauterine growth, Doppler studies of the uterine arteries and on the fetal circulation or presence of CTG abnormalities in the first stage of labor. For the maternal component, duration of the first stage of labor, major chronic conditions, objective assessment of intrapartum treatments and procedures may be all explored in future models and research.

The extent to which our result may be generalizable to premature deliveries remains to be determined with further studies.

This study shows an important clinical concept, described by our group before and confirmed today with new methodology and dataset: identical CTG tracings may be associated with divergent risks of MA in different patients according to maternal, fetal, and labor characteristics. The risk of MA should always be considered even if the CTG is nonpathological, particularly in prolonged 2STGs and when other risk factors are concomitant. In presence of pathological CTG the effect of time should be taken in great consideration, given the higher rate of risk increase overtime in the 2STG. The expected incidence of MA in the general population may be suggested as a possible risk cutoff beyond which expert medical advice and possible corrective interventions are recommendable. Therefore, we believe that this study poses the basis for future research on this topic, potentially leading to more reliable risk assessment in labor ward. This field of research may include reassessment of timing for preventive measures and intrapartum obstetric interventions.

We believe that this study is a further step forward towards personalized and precision medicine in labor ward but we acknowledge that the retrospective nature of the study represents a limit for the estimation of a patient-specific risk for MA, that remains a gold standard of the intrapartum care.

## Supplementary Information

Below is the link to the electronic supplementary material.

Supplementary file1 (TIFF 75 KB)

## Abbreviations

FHR: Fetal heart rate
2STG: 2Nd stage of labor
CTG_RED: Pathologic intrapartum CTG requiring expedited delivery
MA: Umbilical cord metabolic acidemia at birth
CSH: Cause-specific hazard
CIF: Cumulative incidence
HR: Hazard ratio
